# Evaluation of Human Body Fluids for the Diagnosis of Fungal Infections

**DOI:** 10.1155/2013/698325

**Published:** 2013-08-01

**Authors:** Parisa Badiee

**Affiliations:** Alborzi Clinical Microbiology Research Center, Namazi Hospital, Shiraz University of Medical Sciences, Zand Avenue, Shiraz 7193711351, Iran

## Abstract

Invasive fungal infections are a major cause of morbidity and mortality in immunocompromised patients. Because the etiologic agents of these infections are abundant in nature, their isolation from biopsy material or sterile body fluids is needed to document infection. This review evaluates and discusses different human body fluids used to diagnose fungal infections.

## 1. Introduction 

Invasive fungal infections (IFI) are increasing in immunocompromised patients due to the improved management of high-risk patients with novel treatment methods. The risk of IFI has been increasing over the last decades mostly because the medical treatments used in specific setting of patients are correlated with severe and prolonged immunosuppression. Although some types of IFI are rare, they are associated with significant morbidity and mortality [1].

The main etiologic agents include yeasts, filamentous fungi, and dimorphic fungi. Common yeasts are predominantly *Candida *spp. including *Candida albicans*, *Candida glabrata*, *Candida kefyr*, *Candida parapsilosis, Candida tropicalis *[2], and *Cryptococcus* spp. Other organisms such as *Trichosporon* spp. can be involved in serious conditions. The filamentous fungi are classified as having either septate (e.g., *Aspergillus* spp.) or aseptate hyphae (Mucorales) [3]. Less common pigmented molds (dematiaceous, that is, darkly pigmented) including *Pseudallescheria* can infect some human organs, especially in the central nervous system (CNS). Finally, the dimorphic fungi are filamentous at 25°C and room temperature and yeast-like in host tissues or when incubated at 35 to 37°C. These fungi, which are true pathogens, include *Blastomyces*, *Histoplasma*, *Coccidioides*, *Paracoccidioides,* and *Penicillium marneffei *and are endemic in specific geographic regions [4–6].

Analysis of all body fluids is essential for the diagnosis of IFI and may be more informative than serum analysis [7]. Invasive diagnostic procedures such as bronchoscopy, thoracoscopy, percutaneous catheter drainage, or open surgery are usually necessary to confirm the diagnosis but are not possible in some critical patients. For pyogenic abscesses, percutaneous catheter drainage is an established technique with a success rate of 80%–90% [8, 9], although in deep systemic mycosis of poorly localized nature, the success rate is reported to be low [10]. 

To date no typical clinical picture has emerged, so the management of infections is critical and the isolation and identification of the fungal pathogen are important for prompt, appropriate therapy. To identify deep-seated IFI and provide appropriate care according to international criteria, isolation of fungi from biopsy specimens [11] or drainage from any focal fluid sample is necessary [8, 9, 12]. 

 In high-risk patients, the isolation of fungi during followup may appear to contradict the patient's physiological and clinical condition, which may not reflect the severity of the infection. Need for early antifungal therapy is still controversial, especially when *Candida* is isolated from peritoneal fluid, urine, or pulmonary specimens [13–16]. The isolation of fungi from more than one specimen of urine, peritoneal fluid, or blood culture is a reliable criterion for systemic IFI [1].

The methods available for the diagnosis of fungal elements in human body fluids include routine mycological methods such as direct microscopic examination and culture in specific fungal media, serological methods such as the latex antigen test, galactomannan, and mannan antigen or antibody detection by ELISA, and molecular methods such as nested PCR, real-time PCR, and PCR ELISA. Determining the susceptibility of the isolated fungi to antifungal agents can help improve the clinical management of systemic mycosis [3, 17]. Early diagnosis with prompt antifungal therapy or even surgery might be warranted to save the patient's life [18]. This review evaluates and discusses different human body fluids that can be used in the diagnosis and management of IFI. 

## 2. Pleural Effusion

Pulmonary fungal infections are difficult to confirm. The isolation of fungi (especially yeasts) as the pathogenic agent is controversial, so determining the prevalence and management of these infections has been difficult. A previous report demonstrated that *Candida *colonization could be found in respiratory samples obtained by bronchopulmonary lavage, endotracheal aspirate, or protected specimen brushing in critically ill patients [19]. One of the criteria for the diagnosis of pulmonary FI is an obvious lung lesion on chest X-ray and isolation of fungi from the pleural effusion or blood. The pleural fluid obtained in many cases of fungal pleuritis [20] is a reliable specimen for the diagnosis of pulmonary infections. Pulmonary cryptococcal infection is identified by microscopic examination or positive cryptococcal antigen in percutaneous needle aspiration fluid [18]. For the diagnosis of *Aspergillus* fungus ball, surgical specimens from the pleural cavity have been used, but the value of pleural fluid is not known [21].

## 3. Bronchoalveolar Lavage Fluid

Pulmonary fungal infection, especially pulmonary aspergillosisis is routinely diagnosed by examination of bronchoalveolar lavage fluid (BAL). There are series of recommendations for performing BAL fluid, according to European Respiratory Society [22, 23]. 

Conventional mycological techniques like culture and histological examination of BAL fluid are the most commonly used ones for the diagnosis of these infections and the detection of the fungal cell wall antigen can be performed by galactomannan (GM) test [24] and PCR [25] in BAL samples. The sensitivity and specificity of galactomannan Ag test can vary in serum and BAL fluid and they are usually lower in BAL than in serum [24]. According to Musher et al., the sensitivity and specificity of the GM EIA in BAL fluid with positive culture result for *Aspergillus* were 61% and 98% with an index cutoff 1.0 and 76% and 94% with an index cutoff of 0.5, respectively [26]. The sensitivity and specificity of qPCR assay in BAL fluid were 67% and 100%, respectively [26]. 

As oxygenation is likely to deteriorate during the BAL collection, there are difficulties in performing BAL fluid collection in some critical patients like hematology patients with complication rates approaching 15% [27] and patients with borderline oxygenation who require elective preprocedure intubation and ventilation. 

## 4. Peritoneal Fluid

For some patients a permanent vascular access cannot be used, and peritoneal dialysis is performed instead. In such patients, fungal peritonitis [28] is one of the most serious complications. This infection is a rare but potentially fatal complication. Fungal peritonitis accounted for 3.6% [29] and from 5% to 22% [30, 31] of all peritonitis episodes. The mortality rate was from 20% to 30% in one study [32] and 60% to 70% in another [33]. However, in some areas the rates can be much higher, and peritonitis is associated with high rates of morbidity and mortality.

Direct microscopic examination of the peritoneal fluid was useful for confirming suspected IFI in 60% of patients in one study [29]. In patients in the intensive care unit with peritonitis, grade C scores (at least three risk factors) predicted yeast isolation from peritoneal fluid with 84% sensitivity, 50% specificity, 67% positive predictive value, 72% negative predictive value, and an overall accuracy of 71% [34]. About 43.4% of the patients with perforated peptic ulcer [35] had positive peritoneal fluid fungal culture for yeast, including *Candida *spp. The agents responsible for peritonitis according to Martos et al. were *Candida parapsilosis *(4), *C. albicans *(2), *C. tropicalis *(1), *C. glabrata *(1), *C. famata *(1) and *Fusarium oxysporum* (1) [29]. 

## 5. Urine

Candiduria is clearly not a disease and is common in hospitalized patients. Clinical findings vary and can include asymptomatic candiduria (previously healthy patients, predisposed outpatients, or predisposed inpatients), symptomatic candiduria (cystitis, pyelonephritis, prostatitis, epididymo-orchitis, or urinary tract fungus balls), and clinically unstable candiduria [36]. Most patients are asymptomatic. *C. albicans *is the yeast most commonly isolated from urine, accounting for 50% to 70% of the isolates in various studies [37–39]. 

Samples should be collected with the clean-voided urine culture method. Candiduria may occur due to contamination of urine during sampling with perineal flora, especially in older women or when vaginal discharge is present. In these cases, sampling must be repeated and it is often necessary to obtain the urine specimen by sterile bladder catheterization. If the second specimen culture yields no yeasts, contamination can be assumed and no further diagnostic procedures are needed. 

Once contamination is ruled out, colonization of the bladder, perineum, or indwelling urinary catheter must be considered. To verify infection from colonization, the number of organisms in the urine must be quantified. The first studies done in 1970s used renal biopsies to establish renal involvement [31, 40]. In patients without a catheter or with a short indwelling catheter, proven renal infection was found with as few as 10 000 to 15 000 yeasts/mL and as many as 40 000 yeasts/mL in the urine [41]. In addition, criteria such as pyuria, the presence of pseudohyphae [42], and the finding of casts [43, 44] in the urine were considered to distinguish between colonization and urinary tract infection, but these features are of limited practical use in clinical terms. Species such as *Candida glabrata *naturally cannot produce pseudohyphae, and *C. albicans *can be induced to form pseudohyphae by varying pH and nutrient conditions. It was also thought that antibody-coated yeasts in the urine could be used as a marker for infection [45, 46]. 

Some special disorders such as acquired or congenital disturbances of urine flow, occult diabetes mellitus, genitourinary structural abnormalities, bacterial infections, diminished renal function, structural abnormalities of the kidney, and metabolic abnormalities merit particular attention because they are the predisposing factors for candiduria [47, 48]. Candiduria may be due to hematogenous seeding of the kidney cortex in the course of disseminated candidiasis [37]. Experimental studies of hematogenous renal candidiasis in animal models indicated that any concentration of *Candida *spp. in the urine was significant for renal involvement [49]. Even if infection of the urinary tract by *Candida* spp. can be confirmed, surprisingly, physicians do not always follow the patients and antifungal therapy is not always warranted [37–39]. This issue needs to be addressed in further studies of the patients with candiduria. 

## 6. Pericardial Effusion

Fungal pericarditis occurs mainly in immunocompromised patients [50] due to endemic fungi such as *Histoplasma *and *Coccidioides,* opportunistic fungi (*Candida*, *Aspergillus*), and semifungi including *Nocardia* and *Actinomyces* [51–53]. The clinical picture of fungal myocarditis comprises of the full spectrum of pericardial diseases [54]. Fungal pericarditis is diagnosed mainly by staining and culturing pericardial effusion or tissue samples. The samples must be analyzed promptly and should undergo Gram, acid-fast, and fungal staining, followed by cultures [55]. Molecular assays can serve as a reliable method for the diagnosis of fungal endocarditis [56].

## 7. Blood

Diagnostic methods for IFI in the blood vary in sensitivity and specificity. Blood culture for the diagnosis of filamentous fungi is not specific and for *Candida *spp., with a sensitivity about 50% [57, 58]. Candidemia is diagnosed when *Candida *spp. are isolated from at least one blood culture [59]. The mortality rates for candidemia range from 5% in intensive care units in the USA to 71% in liver transplant recipients [60].

Serological methods such as the beta glucan assay in serum may be useful for identifying IFI by all etiologic agents except *Cryptococcus* and *Zygomycetes*, with sensitivity rates of 64% to 77%, although specificity may be decreased in patients with certain concurrent bacterial infections [61, 62]. The serum galactomannan assay was found to have 95% sensitivity and specificity for invasive *Aspergillus *infections [63], and the serum mannan assay had a 67% specificity and 90% sensitivity for the diagnosis of systemic candidiasis in patients with fungemia [64, 65].

Molecular methods have recently been used to detect systemic IFI in serum and whole blood samples. The panfungal PCR assay [61] detects the small-subunit rRNA gene sequence of major fungal organism groups, with a sensitivity of 80% and a specificity of 95.6% [66]. Other molecular methods include PCR ELISA, nested and real-time PCR, which are used to detect fungal DNA in blood samples. These methods can improve the monitoring of the patients after antifungal therapy [67, 68]. In patients with IFI who respond to antifungal therapy, PCR ELISA assays became negative after 14 days of treatment and remained positive in patients who did not respond [69]. The other advantage of molecular methods for blood samples, especially in serial sampling, is the earlier appearance of positive PCR findings compared to other diagnostic methods based on radiological symptoms. Culture methods can take 8–10 days, whereas PCR can yield results in 4-5 hours. In patients who were followed weekly, the PCR findings became positive before clinical manifestations and radiological findings appeared. In other words, the PCR results became positive during the incubation period of the infection [66, 70, 71].

## 8. Cerebrospinal Fluid

Many fungi can cause CNS infection in high-risk patients. Infection in immunocompetent patients has also been reported [72]. Among patients with hematologic malignancies, CNS disease accounts for 9.4% of filamentous IFI [73]. The diagnosis is based on the analysis of cerebrospinal fluid (CSF). In adult normal CSF, the white blood cell (WBC) count may be as high as 5/mL with a predominance of lymphocytes. In normal CSF the glucose concentration is from 50% to 60% of serum values [74].

In patients with fungal meningitis infection, the WBC count is variable and lymphocytic pleocytosis (*Cryptococcus*, *Candida*) is present together with a predominance of neutrophils (*Aspergillus*, *Blastomycosis*) or eosinophilia (*Coccidioides*) [75]. 

On direct microscopic examination, hyphae or pseudohyphae can occasionally be seen in *Candida* or other infections. Indian ink should be used for the diagnosis of *Cryptococcus neoformans*. Fungal cultures are mostly positive in *C. neoformans* and candidal meningitis and are less frequently positive in other IFI [76]. Larger volumes of CSF obtained with repeated lumbar puncture can increase the chances of positive culture results.

Glucose and protein concentrations in CSF are the sensitive indicators of CNS pathology. The physician should know the normal reference range in each region because the measurements are technique dependent and the normal range varies depending on laboratory procedures. In the normal population, CSF glucose is about two thirds of the serum glucose concentration and does not exceed 300 mg/dL regardless of serum levels. The adult range of CSF protein concentration reaches 18 to 58 mg/dL between 6 and 12 months of age [77]. In fungal brain infections, CSF glucose is generally low and protein is generally high, with exceptionally high levels in cryptococcal infections [74]. 

The sensitivity and specificity rates for CSF antigen testing are above 90% for *Cryptococcus* and *Histoplasma *[74, 75] and the galactomannan CSF assay for the diagnosis of invasive aspergillosis has been investigated [63]. In addition, PCR analyses can indicate the presence of DNA from different fungal species in CSF [78].

## 9. Synovial Fluid 

Fungal joint infection due to yeast and filamentous fungi is rare and its diagnosis and treatment can be challenging even for skilled doctors. The etiologic agents reported most frequently are *Candida *and *Aspergillus* spp. In invasive *Aspergillus* infections, dark fluid is aspirated from the joint. In joints with *Aspergillus fumigatus* infection, synovial fluid analysis showed cell count between 7300 and 128 000 cell/mL, predominantly neutrophils [79].

In recent years the incidence of periprosthetic joint infection (total knee replacement) has increased. In one study, most fungal knee infections were caused by *Candida *species (80%), of which *C. parapsilosis* was the most common (50%) [80]. The definitive diagnosis and identification of the etiologic agent were yielded by aspiration of synovial fluid in most cases, but in some cases intraoperative and tissue biopsy culture were needed [81].

## 10. Saliva 

Saliva is the first defense against the many pathogenic organisms in oral cavity. With regard to IFI, the most important role of saliva is defense against pathogenic fungi with special enzymes. *Candida albicans* is the most important fungal species in the oral cavity. The primary mechanism of salivary defense is secretory immunoglobulin, which inactivates fungi via binding and/or agglutination of the microorganisms [82]. Other defense mechanisms have also been hypothesized, such as lactoferrin, lysozyme, and histamine, particularly histatin-5, which can bind to the surface of heat shock protein homologues of *C. albicans* and lead to internalization and death of this organism [83]. The clinical signs of oral candidiasis have been investigated by counting *Candida* organisms in saliva [84], and a sensitive PCR detection system has been used to diagnose *Pneumocystis carinii* pneumonia [85]. 

Tables 1 and 2 presented the use of body fluid and diagnostic methods for the detection of opportunistic fungal infections in body fluids. 

## 11. Conclusions 

The examination of human body fluids is necessary to diagnose IFI, even though invasive methods may be needed to obtain samples for analysis. Routine laboratory tests for the diagnosis of FI include urinalysis and blood analysis. The second line for the diagnosis of systemic mycosis is the CSF. Analyses of both CSF and serum can improve the accuracy of the diagnosis. Other human body fluids should be obtained and analyzed for IFI according to specific conditions.

## Figures and Tables

**Table 1:** Use of body fluid for the detection of fungal infections.

Site of infection	Sample	Most Etiologic fungal agents
Systemic infection	Blood	*Candida *spp., *Aspergillus* spp., *Cryptococcus* spp.

Urinary tract and systemic infection	Urine	*Candida *spp. especially *C. albicans*, * Cryptococcus* spp.

Lung	Pleural effusion, Bronchoalveolar lavage fluid	*Candida* spp., *Aspergillus* spp.,* Cryptococcus* spp., Zycomycetes fungi family

Peritoneum	Peritoneal	*Candida* spp., and rare filamentous fungi like *Fusarium oxysporum or Aspergillus* spp.

Joint	Synovial fluid	*Candida* spp., *Aspergillus fumigatus *

Heart	Pericardial effusion	Endemic fungi such as *Histoplasma* and *Coccidioides* or opportunistic fungi like *Candida*, *Aspergillus* and semi-fungi including *Nocardia* and *Actinomyces *

Central Nervous Infection	Cerebrospinal fluid	*Cryptococcus neoformans*, *Candida* spp., *Aspergillus* spp., *Blastomyces dermatitis*,* Pseudallescheria* and *Histoplasma capsolatum *

Oral	Saliva	*Candida* spp.

**Table 2:** Diagnostic methods for the detection of opportunistic fungal infections in body fluids.

Etiologic agents	Diagnostic methods
Aspergillosis	*Pleural effusion or Bronchoalveolar lavage fluid *
	(i) Direct examination, cultures, histopathologic demonstration
	(ii) Galactomannan antigen detection, Beta-D-glucan assay*, Polymerase chain reaction
	*Serum *
	(i) *Aspergillus* antibody test (precipitins) for chronic pulmonary aspergillosis
	(ii) *Aspergillus* IgG antibodies, *Aspergillus* IgE test (precipitins)
	(iii) Galactomannan and beta-D-glucan levels double sandwich enzyme-linked immunosorbent assay (ELISA)
	(iv) 1,3-Beta-D-glucan

Candidiasis	*Blood, CSF, Urine, or other body fluid *
	(i) Isolating a *Candida* species from multiple or repeat cultures
	(ii) Beta-D-glucan and other antigen and metabolite assays
	(iii) Polymerase chain reaction

Cryptococosis	*Cerebrospinal fluid, Blood or Serum, BAL, Urine *
	(i) India ink smear
	(ii) Culture
	(iii) latex agglutination test or enzyme linked immunosorbent for Cryptococcal antigen testing

Mucormycosis	*Sterile body fluid from site of infection *
	(i) Histopathopathologic examination
	(ii) Culture

Other fungal infection	*Sterile body fluid from site of infection *
	(i) Histopathopathologic examination
	(ii) Culture and isolation of fungal species in cultures of involved biologic materials

*1,3-Beta-D-glucan, a cell wall composition of many fungi, may be positive in patients with a variety of invasive fungal infections, it is typically negative in patients with mucormycosis or cryptococcosis.
